# Treatment-related differences in quality of life and psychological distress among patients with hepatocellular carcinoma: A cross-sectional analysis

**DOI:** 10.1017/S1478951526101758

**Published:** 2026-02-05

**Authors:** Yi-Tseng Tsai, Zan-Ting Lu, Hsin-Yu Kuo, Ya-Han Yang, Yi-Jing Tsai, Kun Feng Tsai, Wen-Chun Liu

**Affiliations:** 1 School of Nursing, China Medical University, Taichung, Taiwan; 2 Department of Nursing, An Nan Hospital, China Medical University, Tainan, Taiwan; 3 Department of Internal Medicine, National Cheng Kung University Hospital, College of Medicine, National Cheng Kung University, Tainan, Taiwan; 4 Department of Nursing, Chang Gung University of Science and Technology, Chiayi, Taiwan; 5 Department of Internal Medicine, An Nan Hospital, China Medical University, Tainan, Taiwan; 6 Department of Nursing, National Tainan Junior College of Nursing, Tainan, Taiwan

**Keywords:** Depression, hepatocellular carcinoma, quality of life, radiofrequency ablation, symptom distress

## Abstract

**Objectives:**

Hepatocellular carcinoma (HCC) is associated with high mortality and imposes substantial symptom and psychological burdens; however, the impact of different treatment modalities on quality of life (QoL) and mental health remains underexplored. This study aimed to examine the associations among symptom distress, depression, and QoL across various HCC treatments.

**Methods:**

A cross-sectional study was conducted with 101 inpatients at a regional hospital in Taiwan (October 2020–December 2021). Patients received hepatic resection (HR), radiofrequency ablation (RFA), transarterial chemoembolization (TACE), hepatic arterial infusion chemotherapy (HAIC), or immunotherapy (IT). Data were collected using the European Organization for Research and Treatment of Cancer Quality of Life Questionnaire Core-30 (EORTC QLQ-C30), the Hospital Anxiety and Depression Scale (HADS), and the Brief Symptom Rating Scale (BSRS).

**Results:**

RFA patients reported better functional scores (96.13 ± 7.55) and lower HADS scores (18.31 ± 4.92) than those treated with TACE, HAIC, or IT (function: 87.77 ± 17.77; HADS: 23.26 ± 7.66). These differences may reflect earlier disease stage and better baseline health in RFA recipients. Older age and advanced stage were associated with poorer global health (*p* < 0.05), while female gender (*β* = − 7.38, *p* = 0.014) and disease recurrence (*β* = − 6.48, *p* = 0.019) were associated with lower functional status.

**Significance of results:**

Treatment type, disease stage, and demographics significantly shape QoL and mental health in HCC patients. Minimally invasive therapies like RFA may preserve QoL in early-stage disease, while invasive or palliative treatments necessitate greater psychosocial support.

## Introduction

Hepatocellular carcinoma (HCC) is the most common type of primary liver cancer and a major malignancy worldwide (Bray et al. 2018; McGlynn et al. 2021). Significant risk factors include hepatitis B and C viruses, high alcohol consumption, and liver cirrhosis (Singal et al. 2020). With a high mortality rate, HCC ranks fourth among the leading causes of cancer-related deaths worldwide (Bray et al. 2018; Singal et al. 2020). The five-year survival rate for patients with HCC is under 20% globally due to the often-asymptomatic nature of early-stage disease, leading to delayed diagnoses (Laube et al. 2021).

The quality of life (QoL) of patients with HCC is significantly affected by poor prognosis, chronic liver disease, and the severity of symptoms in later stages (Fan et al. 2010). Patients with HCC commonly experience physiological symptoms such as pain, fatigue, and loss of appetite, which considerably affect their daily functioning and overall well-being (Bruix et al. 2011; Tung-Ping Poon et al. 2000). Additionally, they are prone to psychological issues, particularly depressive symptoms, which further deteriorate their QoL (Fan et al. 2010; Laube et al. 2021). Ferrell’s cancer-specific QoL model, which includes physical, psychological, social, and spiritual well-being, is especially relevant for patients with HCC (Ferrell et al. 1995). QoL is a critical measure of healthcare effectiveness, particularly when curing HCC is not feasible (Firkins et al. 2021). Importantly, QoL holds independent prognostic value for survival outcomes in patients with HCC (Bonnetain et al. 2008). Considering the terminal nature of HCC, comprehensive QoL assessments are essential for effective patient care (Laube et al. 2021).

Patients with HCC are especially vulnerable to psychological distress due to their cancer diagnosis and treatment, compounded by chronic liver disease (Graf and Stengel 2021; Hernaez et al. 2022; Tan et al. 2022). Moderate-to-severe depression or anxiety is reported by 15–20% of cirrhosis patients, with a higher likelihood observed among women, racial and ethnic minorities, and individuals with lower socioeconomic status (Hernaez et al. 2022). Moreover, factors such as alcohol, substance use, and obesity, which are risk factors for chronic liver disease, can elevate the risk of mental health issues (Grant et al. 2004; McHugh and Weiss 2019; Perry et al. 2021). By contrast, most patients are ineligible for curative treatments such as liver resection or transplantation at diagnosis (Fan et al. 2010; Firkins et al. 2021). Non-curative treatments, including transarterial chemoembolization, radiotherapy, and chemotherapy, can prolong survival but have varying impacts on patients’ physiological and psychological health, influencing overall QoL (Laube et al. 2021). Surgical treatments pose significant risks and may cause physical recovery challenges and psychological stress. Chemotherapy, often used for advanced HCC, can slow disease progression but often causes severe side effects, which in turn contribute to emotional distress and decreased QoL (Palmer 2008). Although radiotherapy can control tumor growth and alleviate symptoms, it causes side effects, such as skin irritation and fatigue, impacting patients’ well-being and mental health (Kudo et al. 2018). Targeted therapies, such as using sorafenib, have fewer side effects than traditional chemotherapy; however, issues such as hand-foot syndrome, diarrhea, and hypertension can occur, which affect patients’ physical health and psychological comfort (Cheng et al. 2009; Firkins et al. 2021).

Although many studies have examined factors related to symptoms and QoL in patients with liver cancer, few have specifically explored the impact of various treatments on depression and symptom distress. Furthermore, no consensus currently exists on the critical factors influencing QoL in patients with liver cancer, particularly in the context of various treatment modalities. This study aimed to bridge this gap by investigating symptom distress, depression, and QoL in patients with HCC. We conducted a comprehensive analysis to identify key demographic and disease-related factors, such as age, gender, disease stage, and recurrence, which significantly impact patient well-being. These findings provide valuable insights into how different treatments and clinical factors affect physical and psychological outcomes, thus offering a clearer understanding of the factors that most profoundly affect QoL in patients with HCC.

## Methodology

### Patient enrollment and study design

This cross-sectional study was conducted between October 27, 2020, and December 31, 2021, following approval from the Institutional Review Board (IRB) of XX Hospital, XX University, Taiwan (Approval No. XXXX107-RECXXX; approved on October 27, 2020). The objective was to examine the relationships among symptom distress, depression, and QoL in patients diagnosed with HCC. A purposive sampling strategy was adopted to recruit 101 inpatients from the oncology and hepatology wards of a regional teaching hospital in southern Taiwan. Eligible participants were identified and approached by two trained research nurses who were independent of the clinical care team. These nurses screened patients according to predefined criteria, explained the study procedures, and obtained verbal informed consent. Inclusion criteria were: (1) age ≥ 18 years; (2) confirmed diagnosis of HCC; (3) awareness of their diagnosis; and (4) receipt of either surgical or non-surgical treatment for HCC. Exclusion criteria included esophageal variceal bleeding, gastrointestinal bleeding, hepatic encephalopathy, alcohol withdrawal syndrome, impaired consciousness, or no prior HCC-specific treatment. The diagnosis of HCC was confirmed through diagnostic imaging (computed tomography or magnetic resonance imaging) or a liver biopsy, as documented in the medical records. Patients underwent a range of treatments, including HR, RFA, TACE, HAIC, IT, and other adjuvant modalities. Symptom distress, depression, and QoL were assessed after treatment (details are provided in the Supplementary Materials).

A priori sample size estimation was conducted using G*Power 3.1 software for a linear multiple regression model. Assuming a medium effect size (*f*^2^ = 0.15), an α level of 0.05, statistical power (1 − *β*) of .80, and inclusion of up to five predictors (e.g., symptom distress, depression, treatment type, age, and disease duration), the minimum required sample size was calculated to be 92 participants. To account for potential attrition or incomplete responses, a target sample size of 100 was established. Ultimately, 101 eligible participants were enrolled and completed the study. Given the minimal risk nature of the study and the use of de-identified data, the Institutional Review Board granted a waiver of written informed consent. Nevertheless, verbal informed consent was obtained from all participants before data collection to ensure voluntary participation.

### Data collection and assessment tools

Data were collected through face-to-face structured interviews conducted in private hospital rooms when participants were medically stable following treatment. Interviews were conducted within 3 to 10 days post-treatment, depending on individual clinical condition and discharge schedule. Each interview lasted approximately 20–30 minutes and was conducted by the same research nurses, who had received standardized training in administering measurement tools. During the interview, participants completed three validated instruments assessing symptom distress, depressive symptoms, and QoL. Responses were recorded on paper forms during the interviews and subsequently entered a secure, password-protected electronic database by the research team.

Assessment data were collected using a structured questionnaire that included sections for demographic information, the European Organization for Research and Treatment of Cancer Quality of Life Questionnaire Core-30 (EORTC QLQ-C30) (Fayers et al. 2002), the Taiwanese version of the Hospital Depression and Anxiety Scale (HADS) (Annunziata et al. 2020), and the brief symptom rating scale (BSRS-5) (Lee et al. 2003). These questionnaires have been previously published, and an English version is available as the supplementary material. The EORTC QLQ-C30 questionnaire comprises five functional scales (i.e., physical, role, cognitive, emotional, and social), three symptom scales (i.e., fatigue, pain, and nausea/vomiting), several single items, and a global health and QoL scale. The HADS is a validated tool used to assess both anxiety and depressive symptoms. It consists of 14 items, with 7 items each for assessing anxiety and depression. Each item is scored on a scale from 0 to 3, with total scores for each subscale ranging from 0 to 21. Higher scores indicate more severe symptoms of anxiety or depression. The BSRS-5 is a brief psychological distress screening tool that assesses five common domains of mental distress: anxiety, depression, hostility, interpersonal sensitivity/inferiority, and insomnia. Each item is rated on a 0–4 scale, with higher total scores reflecting more severe overall psychological distress. Details regarding the instruments, scoring methods, and data management procedures are provided in the Supplementary Materials.

### Statistical analysis

Data were analyzed using SPSS 22.0. Descriptive statistics (percentages, means ± SD) summarized patient characteristics. Categorical variables were compared using the chi-square or Fisher’s exact test, while continuous variables were analyzed with Student’s *t*-test or the Kruskal–Wallis test. Independent *t*-tests and one-way ANOVA assessed mean differences across demographic groups. Pearson correlation analysis examined relationships among symptom distress, depression, and QoL. A *p*-value < 0.05 was considered statistically significant.

## Results

### Demographic and disease characteristics of patients with HCC

This study included 101 HCC patients receiving HR, TACE, HAIC, IT, RFA, and other treatments (Table 1). The average age was 66.62 years, with a male predominance (72.3%). HBV (50.5%) was the most common infection, followed by HCV (16.8%). Patients with early-stage HCC (I–II) were more likely to receive curative treatments, such as HR and RFA. In contrast, those with advanced-stage HCC (III–IV, 38.6%) predominantly underwent palliative treatments, including TACE, HAIC, and IT. Furthermore, treatment response also differed significantly (*p* < 0.001). Complete response (CR) was observed in 45.5% of patients, particularly those undergoing HR (85.7%) and RFA (62.5%). Progressive disease (PD) occurred in 30.7%, mainly in patients receiving TACE, HAIC, or IT. Stable disease (SD) was noted in 20.8%, indicating cases where the disease remained unchanged.

**Table 1.** Demographic and disease characteristics of HCC patients (*N* = 101)

Variables	All patients (*n* = 101)	HR (*n* = 14)	TACE/HAIC/IT (*n* = 35)	RFA (*n* = 32)	Others (*n* = 20)	*p-*Value
Age (year, Mean ± SD)	66.62 ± 10.31	66.63 ± 12.54	66.88 ± 9.68	64.91 ± 8.82	69.48 ± 11.78	0.635
<59 (%)	24 (23.8)	5 (35.7)	7 (20.0)	8 (25.0)	4 (20.0)	
60–69 (%)	37 (36.6)	3 (21.4)	12 (34.3)	15 (46.9)	7 (35.0)	
70–79 (%)	29 (28.7)	4 (28.6)	13 (37.1)	7 (21.9)	5 (25.0)	
> 80 (%)	11 (10.9)	2 (14.3)	3 (8.6)	2 (6.2)	4 (20.0)	
Gender (Male, %)	73 (72.3)	10 (71.4)	29 (82.9)	21 (65.6)	13 (65.0)	0.362
Education (< High school, %)	77 (76.2)	10 (71.4)	27 (77.1)	22 (68.8)	18 (90.0)	0.351
Marital status						0.764
Unmarried (%)	7 (6.9)	1 (7.2)	3 (8.6)	2 (6.3)	1 (5.0)	
Married (%)	75 (74.3)	10 (71.4)	25 (71.4)	27 (84.4)	13 (65.0)	
Divorced (%)	3 (3.0)	0 (0.0)	2 (5.7)	0 (0.0)	1 (5.0)	
Separated/ widowed (%)	16 (15.8)	3 (21.4)	5 (14.3)	3 (9.4)	5 (25.0)	
Religion						0.836
None (%)	17 (16.8)	2 (14.3)	6 (17.1)	5 (15.6)	4 (20.0)	
Taoism (%)	66 (65.3)	10 (71.4)	24 (68.6)	18 (56.3)	14 (70.0)	
Buddhism (%)	17 (16.8)	2 (14.3)	5 (14.3)	8 (25.0)	2 (10.0)	
Christianity (%)	1 (1.0)	0 (0.0)	0 (0.0)	1 (3.1)	0 (0.0)	
Body mass index (BMI)^a^						0.677
Underweight (%)	5 (5.0)	1 (7.2)	1 (2.9)	2 (6.2)	1 (5.0)	
Normal weight (%)	47 (46.5)	5 (35.7)	19 (54.3)	13 (40.6)	10 (50.0)	
Overweight (%)	27 (26.7)	5 (35.7)	10 (28.6)	6 (18.8)	6 (30.0)	
Obesity (%)	22 (21.8)	3 (21.4)	5 (14.3)	11 (34.4)	3 (15.0)	
Income (NTD/month)^b^						0.386
Dependent (%)	66 (65.3)	8 (54.1)	26 (74.3)	17 (53.1)	15 (75.0)	
<37,000 (%)	19 (18.8)	2 (14.3)	4 (11.4)	9 (28.1)	4 (20.0)	
37,001–43,000 (%)	11 (10.9)	2 (14.3)	3 (8.6)	5 (15.6)	1 (5.0)	
≥ 43,001 (%)	5 (5.0)	2 (14.3)	2 (5.7)	1 (3.2)	0 (0.0)	
CCI^c^ comorbidity scoring						0.434
0 (%)	1 (1.0)	1 (7.1)	0 (0.0)	0 (0.0)	0 (0.0)	
1 (%)	2 (2.0)	0 (0.0)	2 (5.7)	0 (0.0)	0 (0.0)	
2 (%)	5 (5.0)	1 (7.1)	2 (5.7)	1 (3.1)	1 (5.0)	
3 (%)	16 (15.8)	2 (14.3)	4 (11.4)	5 (15.6)	5 (25.0)	
≥4 (%)	77 (76.2)	10 (71.5)	27 (77.2)	26 (81.3)	14 (70.0)	
Hepatitis classification						0.902
Non-viral hepatitis (%)	33 (32.7)	6 (42.9)	11 (31.4)	9 (28.1)	7 (35.0)	
HBV infected (%)	51 (50.5)	7 (50.0)	17 (48.6)	18 (56.3)	9 (45.0)	
HCV infected (%)	17 (16.8)	1 (7.1)	7 (20.0)	5 (15.6)	4 (20.0)	
TNM stage^d^						< 0.001
I (%)	37 (36.6)	7 (50.0)	6 (17.1)	19 (59.4)	5 (25.0)	
II (%)	25 (24.8)	6 (42.9)	8 (22.9)	9 (28.1)	2 (10.0)	
III (%)	9 (8.9)	0 (0.0)	5 (14.3)	0 (0.0)	4 (20.0)	
IV (%)	30 (29.7)	1 (7.1)	16 (45.7)	4 (12.5)	9 (45.0)	
Recurrence (yes, %)	38 (37.6)	2 (14.3)	15 (42.9)	12 (37.5)	9 (45.0)	0.249
Treatment response in solid tumors^e^						< 0.001
PD (%)	31 (30.7)	1 (7.1)	17 (48.6)	7 (21.9)	6 (30.0)	
SD (%)	21 (20.8)	1 (7.1)	7 (20.0)	5 (15.6)	8 (40.0)	
PR (%)	3 (3.0)	0 (0.0)	3 (8.6)	0 (0.0)	0 (0.0)	
CR (%)	46 (45.5)	12 (85.8)	8 (22.8)	20 (62.5)	6 (30.0)	

a *Notes:* Body mass index (BMI): BMI is calculated as weight in kilograms divided by height in meters squared (kg/m^2^). The classification of body weight based on BMI is as follows: Underweight, A BMI less than 18.5; Normal weight, A BMI between 18.5 and 24.0; Overweight, A BMI between 24.0 and 27.0; Obesity, A BMI of 27.0 or higher.

b New Taiwan dollar (NTD): Currency conversion: 1USD = 29.34NTD.

c Charlson Comorbidity Index (CCI): The CCI uses disease diagnosis codes to classify different types of chronic diseases and assigns weights based on disease severity (weights range from 1 to 6 points, with higher points indicating greater severity).

d TNM Staging System: TNM staging is based on the guidelines of American Joint Committee on Cancer (AJCC) and the International Union Against Cancer (UICC).

e Treatment refers to the primary therapeutic approach administered to the patient. This includes hepatic resection (HR, *n* = 14), transarterial chemoembolization (TACE, *n* = 25), hepatic arterial infusion chemotherapy (HAIC, *n* = 8), immunotherapy (IT, *n* = 2), radiofrequency ablation (RFA, *n* = 32), and other treatments. Other treatments include symptom management only (*n* = 13), chemotherapy (*n* = 1), radiotherapy (*n* = 2), targeted therapy (*n* = 3), and concurrent chemoradiotherapy (CCRT, *n* = 1). The objective response to treatment is divided into four categories: Complete response (CR), partial response (PR), stable disease (SD), and progressive disease (PD).

### Characteristics of depression, anxiety, and QoL among patients with HCC

The QoL, depression, anxiety, and psychological distress were assessed using the EORTC QLQ-C30, HADS-D, and BSRS questionnaires (Table 2). Patients exhibited high overall functioning (90.56 ± 14.21) but reported moderate global health perceptions (63.02 ± 17.66) and notable symptom burden (8.15 ± 9.47). Role function was the highest (96.68 ± 10.57), while physical function was the lowest (86.18 ± 18.86). Treatment significantly influenced overall function (*p* = 0.038), with RFA patients reporting the highest scores (96.13 ± 7.55), indicating better physical and social functioning compared to those receiving TACE, HAIC, or IT (87.77 ± 17.77). Emotional function also varied significantly, with RFA patients showing better emotional well-being (97.16 ± 6.22, *p* = 0.007).

**Table 2.** Questionnaire outcomes on quality of life (QoL), depression and anxiety, and brief symptom rating scale (BSRS) scores in HCC patients after treatment

Variables	All patients (*n* = 101)	HR (*n* = 14)	TACE/HAIC/IT (*n* = 35)	RFA (*n* = 32)	Others (*n* = 20)	Range	*p-*Value
QoL (EORTC-QLQ-C30)							
Global health status	63.02 ± 17.66	62.57 ± 14.04	59.57 ± 18.22	67.81 ± 15.77	61.70 ± 21.09	17–100	0.258
Function	90.56 ± 14.21	91.29 ± 8.81	87.77 ± 17.77	96.13 ± 7.55	86.05 ± 16.23	24–100	0.038*
Physical function	86.18 ± 18.86	87.93 ± 10.23	82.69 ± 23.29	92.25 ± 13.48	81.35 ± 20.55	0 − 100	0.113
Role function	96.68 ± 10.57	98.79 ± 4.54	95.69 ± 13.05	99.47 ± 3.01	92.50 ± 14.75	33–100	0.099
Cognitive function	94.04 ± 14.44	95.21 ± 10.18	93.31 ± 17.21	97.41 ± 10.43	89.10 ± 16.48	17–100	0.236
Emotional function	89.78 ± 14.93	87.50 ± 17.65	86.69 ± 15.82	97.16 ± 6.22	85.00 ± 17.67	33–100	0.007**
Social function	92.93 ± 18.27	94.07 ± 12.33	89.54 ± 24.92	97.94 ± 8.12	90.05 ± 19.01	0 − 100	0.247
Symptom	8.15 ± 9.47	6.29 ± 4.81	9.11 ± 12.05	6.34 ± 7.49	10.65 ± 9.39	0–62	0.329
Fatigue	12.53 ± 13.61	13.36 ± 12.34	15.11 ± 16.58	7.91 ± 9.37	14.85 ± 13.48	0 − 78	0.133
Pain	6.80 ± 13.80	2.43 ± 6.17	9.11 ± 15.88	4.19 ± 13.43	10.00 ± 13.60	0–67	0.203
Nausea and vomiting	3.65 ± 10.19	.00 ± 0.00	3.86 ± 12.27	4.16 ± 10.31	5.05 ± 9.56	0–67	0.525
Dyspnea	13.11 ± 19.97	9.43 ± 15.47	12.29 ± 21.43	13.44 ± 18.57	16.60 ± 22.94	0–100	0.769
Insomnia	8.84 ± 16.17	9.43 ± 15.47	9.49 ± 19.07	7.22 ± 13.86	9.90 ± 15.52	0–67	0.925
Appetite loss	12.16 ± 21.44	7.07 ± 14.05	14.26 ± 25.95	8.28 ± 16.88	18.25 ± 22.87	0–100	0.288
Constipation	5.24 ± 13.03	2.36 ± 8.82	3.77 ± 10.65	6.19 ± 13.09	8.30 ± 18.32	0–67	0.499
Diarrhea	3.92 ± 10.73	7.07 ± 14.05	2.83 ± 9.37	4.13 ± 11.09	3.30 ± 10.16	0 − 33	0.655
Financial difficulties	2.61 ± 8.96	.00 ± 0.00	2.83 ± 9.37	1.03 ± 5.83	6.60 ± 13.54	0–33	0.103
Depression and anxiety (HADS)	21.48 ± 6.88	21.64 ± 5.62	23.26 ± 7.66	18.31 ± 4.92	23.30 ± 7.56	2 − 27	0.013*
Depressed state	10.94 ± 3.76	11.00 ± 3.01	11.86 ± 4.07	9.19 ± 2.93	12.10 ± 4.01	0–16	0.010*
Anxiety state	10.53 ± 3.32	10.64 ± 2.74	11.40 ± 3.71	9.13 ± 2.30	11.20 ± 3.82	2–13	0.028*
Psychological distress (BSRS)	1.55 ± 2.24	1.64 ± 2.06	1.94 ± 2.38	.78 ± 1.39	2.05 ± 2.95	0–10	0.118
Level of emotional distress	1.46 ± 2.00	1.57 ± 1.87	1.83 ± 2.15	.72 ± 1.22	1.90 ± 2.57	0–9	0.085
Suicidal ideation	0.10 ± 0.36	0.07 ± 0.27	0.11 ± 0.40	0.06 ± 0.25	0.15 ± 0.49	0–2	0.836

*Notes*: QoL: Quality of Life as measured by the EORTC QLQ-C30; HADS: Hospital Anxiety and Depression Scale; BSRS: Brief Symptom Rating Scale. Values are presented as mean ± standard deviation. *P*-values indicate the statistical significance of differences between treatment groups. A *p*-value of less than 0.05 was considered statistically significant (*p < 0.05, **p < 0.01). Categories include global health status, function (e.g., physical, role, cognitive, emotional, social), symptom scales (e.g., fatigue, pain, nausea/vomiting, dyspnea, insomnia, appetite loss, constipation, diarrhea, financial difficulties), depression and anxiety (total score, depressed state, anxiety state), psychological distress, level of emotional distress, and suicidal ideation.

Mental health assessments (HADS) revealed mild to moderate depression and anxiety (21.48 ± 6.88), with significant differences across treatment groups (*p* = 0.013 for total score, *p* = 0.010 for depression, and *p* = 0.028 for anxiety) (Table 2). Patients undergoing RFA reported lower depression and anxiety levels compared to those receiving TACE, HAIC, or IT. Psychological distress (BSRS: 1.55 ± 2.24) and suicidal ideation (0.10 ± 0.36) were minimal, with no significant differences among treatment groups.

### Effects of demographic and disease-related factors on QoL, depression and anxiety, and BSRS in patients with HCC

We further investigated the effects of demographic and disease-related factors on QoL, depression and anxiety, and psychological distress in patients with HCC (Table 3). Gender and HCV status significantly influenced symptom distress and psychological distress. Female patients reported higher symptom distress (10.04 ± 12.90 vs. 7.42 ± 7.75, *p* < 0.05) and psychological distress (BSRS scores: 2.18 ± 3.03 vs. 1.32 ± 1.83, *p* < 0.05). Similarly, HCV-positive patients exhibited greater symptom distress (12.82 ± 15.72 vs. 7.20 ± 7.41, *p* < 0.01) and higher BSRS scores (2.71 ± 3.35 vs. 1.32 ± 1.89, *p* < 0.001), while HBV-positive patients had lower symptom distress (6.33 ± 6.46 vs. 10.00 ± 11.55 *p* < 0.05), suggesting different symptom patterns between HBV and HCV cases (Table 3).

**Table 3.** Relationship between demographic attributes, disease characteristics, evaluation of treatment effect, QoL, depression and anxiety, and BSRS in patients with HCC

	QoL (EORTC-QLQ-C30)														
	Global health status			Function			Symptom			Depression and anxiety (HADS)			Psychological distress (BSRS)		
Variables	Mean	SD	*F/t*	Mean	SD	*F/t*	Mean	SD	*F/t*	Mean	SD	*F/t*	Mean	SD	*F/t*
Gender															
Male (*n* = 73)	63.44	16.49	t = 0.38	92.10	10.28	t = 1.33^c^	7.42	7.75	t = −1.00^a^	21.10	6.39	t = −0.89	1.32	1.83	t = −1.41^c^
Female (*n* = 28)	61.93	20.69		86.57	21.06		10.04	12.90		22.46	8.08		2.18	3.03	
Age (years)															
<59 (*n* = 24)	65.38	16.95	F = 1.61	92.54	8.12	F = 0.98	7.17	7.01	F = 0.48	20.50	6.20	F = 0.95	1.42	2.17	F = 0.44
60–69 (*n* = 37)	64.46	19.77		90.97	16.86		9.43	12.64		21.03	7.42		1.54	2.39	
70–79 (*n* = 29)	63.31	16.22		90.93	14.63		7.00	6.88		21.69	6.68		1.41	2.04	
> 80 (*n* = 11)	52.27	12.94		83.91	13.66		9.00	7.89		24.55	6.99		2.27	2.57	
Education															
< High school (*n* = 77)	62.73	17.81	t = −0.30	90.77	13.48	t = 0.26	8.18	8.24	t = .06	21.47	7.13	t = −0.02	1.53	2.21	t = −0.18
≥High school (*n* = 24)	63.96	17.52		89.92	16.64		8.04	12.86		21.50	6.19		1.63	2.39	
Marital status															
Unmarried (*n* = 26)	59.96	17.34	t = −1.03	89.08	11.90	t = −0.62	8.92	8.13	t = 0.48	23.12	6.77	t = 1.42	2.12	2.39	t = 1.49
Married (*n* = 75)	64.08	17.76		91.08	14.97		7.88	9.92		20.91	6.88		1.36	2.17	
Religion															
No (*n* = 17)	62.76	18.22	F = 1.92	91.71	9.26	F = 1.72	7.65	9.47	F = 0.73	21.12	7.11	F = 0.92	1.65	2.50	F = 0.60
Taoism (*n* = 66)	61.56	17.90		88.55	16.47		8.97	10.15		22.11	7.37		1.70	2.32	
Buddhism (*n* = 17)	66.76	14.36		96.71	3.39		5.94	6.29		19.82	4.12		1.00	1.66	
Christianity (*n* = 1)	100	–		100	–		0	–		14	–		0	–	
Income (NTD/month)															
Dependent (*n* = 66)	61.56	16.91	F = 2.27	89.03	16.35	F = 0.96	8.88	10.31	F = 1.52	21.91	7.28	F = 0.53	1.74	2.51	F = 0.96
<37,000 (*n* = 19)	72.00	18.40		95.16	7.36		4.16	5.10		19.68	5.06		.84	1.12	
37,001–43,000 (*n* = 11)	57.55	20.30		92.00	7.59		9.18	9.78		21.45	7.78		1.36	1.50	
>43,001 (*n* = 5)	60.20	9.31		90.20	13.12		11.40	7.45		22.60	6.11		2.20	2.95	
Body mass index (BMI)															
Underweight (*n* = 5)	56.80	9.31	F = 0.46	86.80	10.13	F = 1.15	8.60	5.46	F = 0.86	22.20	5.93	F = 1.32	1.40	1.14	F = 1.49
Normal weight (*n* = 47)	62.09	18.94		88.94	15.63		8.98	9.07		22.68	7.42		1.68	2.29	
Overweight (*n* = 27)	63.37	18.36		90.22	16.14		8.96	12.16		21.17	6.70		2.04	2.72	
Obesity (*n* = 22)	66.00	15.79		95.32	7.19		5.27	6.82		19.23	5.81		.73	1.42	
HBV infected															
No (*n* = 50)	60.40	17.49	t = −1.49	88.00	16.59	t = −1.82	10.00	11.55	t = 1.96^a^	22.14	7.19	t = 0.96	1.88	2.57	t = 1.45
Yes (*n* = 51)	65.59	17.62		93.08	11.01		6.33	6.46		20.82	6.58		1.24	1.84	
HCV infected															
No (*n* = 84)	64.46	17.98	t = 1.85	91.26	13.18	t = 1.10	7.20	7.41	t = −1.44^b^	21.44	6.76	t = −0.11	1.32	1.89	t = −1.65^b^
Yes (*n* = 17)	55.88	14.41		87.12	18.59		12.82	15.72		21.65	7.70		2.71	3.35	
TNM stage															
I (*n* = 37)	64.54	13.68	F = 2.45	94.97	8.20	F =4.13^b^, I > IV	5.49	5.56	F =2.96^a^	18.81	5.01	F =4.30^b^, IV > I	0.86	1.51	F =2.72^a^, IV > I
II (*n* = 25)	68.40	19.20		93.20	8.53		6.84	8.17		21.16	6.07		1.56	2.08	
III (*n* = 9)	51.89	13.29		86.78	11.22		12.22	10.40		24.11	7.08		1.56	1.67	
IV (*n* = 30)	60.00	20.34		84.07	20.95		11.30	12.63		24.23	8.30		2.40	2.97	
Treatment															
HR (*n* = 14)	62.57	14.04	F = 2.07	91.29	8.81	F =3.37^a^, RFA > TACE/HAIC/IT	6.29	4.81	F = 0.86	21.64	5.62	F = 5.13^b^ TACE/HAIC/IT > RFA	1.64	2.06	F = 2.95
TACE/HAIC/IT (*n* = 35)	59.57	18.22		87.77	17.77		9.11	12.05		23.26	7.66		1.94	2.39	
RAF (*n* = 32)	67.81	15.77		96.13	7.55		6.34	7.49		18.31	4.92		0.78	1.39	
Recurrence															
No (*n* = 63)	65.03	16.18	t = 1.48	93.95	7.50	t = 2.67^c^	6.79	7.29	t = −1.67^a^	20.19	5.71	t = −2.27^b^	1.25	2.00	t = −1.65^a^
Yes (*n* = 38)	59.68	19.66		84.95	20.00		10.39	12.04		23.61	8.13		2.05	2.55	
Treatment response															
PD (*n* = 31)	57.52	20.61	F = 1.59	81.77	20.90	F =6.80^c^, SD/CR>PD	12.77	12.86	F = 4.03^b^, PD>CR	25.68	7.74	F = 6.84^c^, PD>CR	2.52	2.59	F = 3.55^a^, PD>CR
SD (*n* = 21)	64.43	15.18		93.19	7.70		7.00	6.40		20.57	5.08		1.43	2.42	
PR (*n* = 3)	61.00	19.05		96.33	4.73		7.67	7.51		21.00	6.56		2.33	3.22	
CR (*n* = 46)	66.22	16.10		94.91	7.24		5.59	6.79		19.09	5.80		0.91	1.59	

*Note*: Independent samples t-tests were used to compare means between two groups, while one-way ANOVA was employed for comparisons across multiple groups. Post hoc analyses were conducted using Scheffe’s test to identify specific group differences. Statistical significance was set at ^a^*p* < 0.05, ^b^*p* < 0.01, and ^c^*p* < 0.001. The objective response to treatment is divided into four categories: Complete response (CR), partial response (PR), stable disease (SD) and progressive disease (PD). BSRS: Brief Symptom Rating Scale. HADS-D: Depression subscale of the Taiwanese version of the Hospital Anxiety and Depression Scale. QoL: Quality of Life.

Both TNM stage and recurrence status significantly impacted patient outcomes. Advanced-stage (stage IV) patients experienced significantly lower functional scores (84.07 ± 20.95 vs. 94.97 ± 8.20, *p* < 0.01), higher depression and anxiety levels (24.23 ± 8.30 vs. 18.81 ± 5.01, *p* < 0.01), increased symptom distress (11.30 ± 12.63 vs. 5.49 ± 5.56, *p* < 0.05), and higher psychological distress (BSRS: 2.40 ± 2.97 vs. 0.86 ± 1.51, *p* < 0.05) compared to early-stage (stage I) patients. Similarly, recurrent HCC patients reported worse functional scores (84.95 ± 20.00 vs. 93.95 ± 7.50, *p* < 0.001) and higher levels of depression and anxiety(23.61 ± 8.13 vs. 20.19 ± 5.71, *p* < 0.01), symptom distress (10.39 ± 12.04 vs. 6.79 ± 7.29, *p* < 0.05), and psychological distress (BSRS: 2.05 ± 2.55 vs. 1.25 ± 2.00, *p* < 0.05) compared to non-recurrent cases (Table 3).

Comparing treatment modalities, RFA patients showed the highest functional scores (96.13 ± 7.55), significantly outperforming those treated with TACE, HAIC, or IT (87.77 ± 17.77, *p* < 0.05) (Table 3). In terms of mental health, they also reported the lowest depression and anxiety levels (HADS: 18.31 ± 4.92 vs. 23.26 ± 7.66, *p* < 0.01), suggesting that RFA’s minimally invasive nature preserves physical and social functioning while reducing emotional distress. In contrast, TACE, HAIC, and IT were associated with greater psychological burden.

Regarding treatment response, patients with PD had significantly lower functional scores (81.77 ± 20.90) than those with SD (93.19 ± 7.70) or CR (94.91 ± 7.24) (*p* < 0.001). PD patients also reported markedly higher symptom distress and depression and anxiety scores compared to CR patients (*p* < 0.01 and *p* < 0.001, respectively). In addition, PD patients had elevated psychological distress (BSRS: 2.52 ± 2.59 vs. 0.91 ± 1.59, *p* < 0.05), reinforcing the link between poor treatment outcomes and increased mental health burden (Table 3).

### Correlation analysis of psychological distress and QoL in patients with HCC

Our analysis identified significant correlations between QoL, depression and anxiety (HADS), and psychological distress (BSRS) in HCC patients (Table 4). Better functional abilities were associated with higher global health perception (*r* = 0.66, *p* < 0.001), while greater symptom severity correlated with poorer global health (*r* = − 0.63, *p* < 0.001). Depression and anxiety negatively impacted both global health (r = − 0.69, *p* < 0.001) and functional abilities (*r* = − 0.82, *p* < 0.001), highlighting the detrimental effect of psychological distress on physical and social functioning. Furthermore, depression and anxiety scores were strongly linked to symptom burden (*r* = 0.71, *p* < 0.001), emphasizing their role in symptom perception. Psychological distress was also associated with poorer global health (*r* = − 0.60, *p* < 0.001) and reduced functionality (*r* = − 0.81, *p* < 0.001). Moreover, BSRS scores correlated positively with symptom severity (*r* = 0.73, *p* < 0.001) and depression and anxiety (*r* = 0.78, *p* < 0.001), reinforcing the interconnectedness between mental health, physical symptoms, and overall QoL in HCC patients.

**Table 4.** Correlation analysis of depression and anxiety, BSRS, and QoL

	QoL (EORTC-QLQ-C30)			Depression and anxiety (HADS)	Psychological distress (BSRS)
Variables	Global health status	Function	Symptom		
QoL (EORTC-QLQ-C30)					
Global health status	–	–	–	–	–
Function	0.66^a^	–	–	–	–
Symptom	−0.63^a^	−0.78^a^	–	–	–
Depression and anxiety (HADS)	−0.69^a^	−0.82^a^	0.71^a^	–	–
Psychological distress (BSRS)	−0.60^a^	−0.81^a^	0.73^a^	0.78^a^	–

a *Notes*: All correlation coefficients were statistically significant at *p* < 0.001. QoL: Quality of Life. HADS: Hospital Anxiety and Depression Scale. BSRS: Brief Symptom Rating Scale.

In HCC patients undergoing different treatments (HR, TACE/HAIC/IT, and RFA), better functional abilities and lower symptom burden were linked to improved health perceptions, while higher depression, anxiety, and psychological distress were consistently associated with poorer outcomes (Supplementary Tables S1–S3). For HR patients (Supplementary Table S1), functional status strongly correlated with global health (*r* = 0.77, *p* < 0.01). Conversely, a higher symptom burden, along with increased levels of depression and anxiety, was linked to poorer health perceptions and reduced functionality. Psychological distress played a major role, strongly affecting the physical function of these patients (*r* = − 0.90, *p* < 0.001).

In the TACE/HAIC/IT group (Supplementary Table S2), depression and anxiety were closely related to greater symptom burden (*r* = 0.71, *p* < 0.001), reduced functional abilities (*r* = − 0.78, *p* < 0.001), and poorer global health status (*r* = − 0.67, *p* < 0.001). Psychological distress (BSRS) further exacerbated functional decline (*r* = − 0.89, *p* < 0 0.001) and symptom severity (*r* = 0.79, *p* < 0.001), indicating a strong interplay between physical and psychological burdens.

Among RFA patients (Supplementary Table S3), functional status showed a moderate correlation with global health (*r* = 0.54, *p* < 0.01). Depression and anxiety were linked to greater symptom burden (*r* = 0.56, *p* < 0.01) and negatively impacted both functionality (*r* = − 0.74, *p* < 0.001) and global health (*r* = − 0.51, *p* < 0.01). Psychological distress strongly correlated with reduced functional abilities (*r* = − 0.81, *p* < 0.001) and higher symptom burden (*r* = 0.66, *p* < 0.001), underscoring the interconnectedness between mental health, physical symptoms, and overall well-being in RFA patients.

### Factors influencing QoL in patients with HCC

Using the EORTC QLQ-C30 questionnaire, we examined the global health and functional abilities of patients with HCC. The stepwise regression analysis identified age and disease TNM stage as key predictors of QoL (Table 5). Each additional year of age was associated with a 0.37-point decline in global health scores (*p* = 0.031), while stage III patients had significantly lower scores than stage I patients (*β* = − 12.55, *p* = 0.049), underscoring the impact of disease progression on well-being. Although stage IV patients also showed lower QoL scores, the difference was not statistically significant.

**Table 5.** Stepwise regression analysis of predictors of quality of life (EORTC QLQ-C30) focusing on global health status and functional status (*N* = 101)

Variables	B	Standard error	F	95% CI	*p*-value
Global health status					
Age	−0.38	0.17	4.81	−0.70, −0.04	0.031*
Stage					0.046*
I (Ref.)					
II	5.25	4.43	1.40	−3.55, 14.05	0.239
III	−12.55	6.30	3.97	−25.07, −0.04	0.049*
IV	−3.46	4.19	0.68	−11.78, 4.85	0.410
Functional status					
Gender	−7.38	2.94	−2.50	−13.74, −1.50	0.014*
Recurrence	−6.48	2.70	−2.40	−11.84, −1.11	0.019*
Stage					0.004**
I (Ref.)					
II	−0.92	3.31	−0.28	−7.48, 5.65	0.782
III	−9.05	4.80	−1.89	−18.58, 0.48	0.062
IV	−10.74	3.19	−3.37	−17.07, −4.40	0.011*

*Notes*: The table presents the results of stepwise regression analyses identifying significant predictors of quality of life in patients with HCC. For global health status, age and disease stage were significant predictors, with Stage I as the reference category. For functional status, gender, disease recurrence, religious affiliation, and disease stage were significant predictors, with no religious affiliation and Stage I as the combined reference category. A *p*-value of less than 0.05 was considered statistically significant (*p < 0.05, **p < 0.01).

Gender and disease recurrence significantly affected functional status (Table 5). Female patients exhibited significantly lower functional status than males (*β* = − 7.38, SE = 2.94, *p* = 0.014), and recurrence further reduced functional status by 6.48 points (*β* = − 6.48, *p* = 0.019). Disease stage remained a strong predictor, with stage IV patients showing a significant 10.74-point decline in functionality compared to stage I (*β* = − 10.74, SE = 3.19, *p* = 0.011). Although stage III patients also showed a decrease in functional status (*β* = − 9.05, SE = 4.80), this was not statistically significant (*p* = 0.062). No significant difference was observed between stages I and II.

## Discussion

To the best of our knowledge, this study is the first to comprehensively examine QoL in patients with HCC by integrating disease characteristics, treatment effect evaluation, and recurrence status through validated assessment tools, including the QoL assessment (EORTC QLQ-C30), HADS, and BSRS. Our findings highlighted that female patients and those with HCV infection experienced higher symptom burdens and worse QoL. Patients with PD exhibited significantly lower functional scores and increased symptom distress, depression, and anxiety compared to those with SD or CR, reinforcing the substantial impact of psychological distress on patient well-being. Patients who underwent curative and minimally invasive treatments, such as RFA, generally demonstrated better QoL and lower psychological distress than those receiving palliative therapies (e.g., TACE, HAIC, and IT). However, these differences likely reflect the underlying disease stage, as early-stage patients were more likely to receive RFA, whereas advanced-stage patients predominantly underwent palliative treatments. Therefore, treatment modality in this study may act as a proxy for disease stage rather than an independent determinant of QoL. Moreover, age, disease stage, and treatment type emerged as key predictors of QoL, with older age, advanced-stage disease, and recurrence contributing to significantly poorer outcomes. Notably, stage IV patients exhibited the most pronounced decline in functional ability, highlighting the need for targeted interventions to preserve QoL in this vulnerable group.

A key strength of this study is its detailed analysis of treatment-related patterns in mental health and QoL. Patients receiving more invasive treatments, such as TACE, tended to report greater symptom distress, depression, and anxiety, underscoring the need for individualized treatment strategies that take into account disease stage, liver function, and overall health. Consistent with the American Association for the Study of Liver Diseases (AASLD) and the European Association for the Study of the Liver (EASL) guidelines, RFA and HR remain the preferred options for early-stage HCC, with RFA offering the potential to better preserve QoL because of its minimally invasive nature. Our findings are consistent with prior research (Bruix et al. 2011; Firkins et al. 2021), showing that patients treated with RFA generally had more favorable functional and psychological profiles than those undergoing TACE or HAIC. However, these differences are likely attributable in part to the earlier disease stage and better baseline health status of patients receiving RFA.

Building on these observations, it is also important to consider treatment strategies for patients in intermediate and advanced stages of HCC. For intermediate- and advanced-stage HCC, TACE remains a standard treatment (Llovet et al. 2002), but targeted therapies (e.g., sorafenib, lenvatinib) (Cheng et al. 2009; Kudo et al. 2018) and immunotherapies (e.g., PD-1 inhibitors) (Finn et al. 2020) are increasingly important in managing disease progression. Although TACE is effective in controlling tumor growth, previous studies have noted its association with a higher burden on QoL, particularly psychological health (Kudo et al. 2018). In agreement with these observations, our data showed that patients who underwent TACE more frequently reported greater symptom distress, depression, and anxiety. These findings likely reflect, in part, the advanced disease stage and poorer baseline condition of these patients rather than the treatment modality alone. Based on current guidelines and our observations, RFA remains a preferred option for early-stage HCC because it is less invasive and may help preserve QoL. For intermediate and advanced stages, the potential psychological burden of invasive procedures such as TACE highlights the importance of considering targeted therapies or immunotherapies, which are associated with fewer side effects and may help maintain overall well-being (Bruix et al. 2011; Firkins et al. 2021; Kudo et al. 2018).

Beyond treatment-related effects, demographic and clinical characteristics also play a crucial role in shaping QoL outcomes. Our study found that, in addition to treatment modality, age, gender, and disease stage are strongly associated with variations in QoL among patients with HCC. Older patients and those in advanced stages (III and IV) tended to have poorer physical functioning and higher levels of depression and anxiety. Furthermore, female patients more frequently reported psychological distress, highlighting the need for tailored psychological support. These observations are consistent with previous studies. Laube et al. (2021) reported that older and late-stage HCC patients tend to have significantly lower QoL due to more severe symptom burdens and mental health challenges. Similarly, Firkins et al. (2021) emphasized that female patients with HCC are more susceptible to psychological distress, suggesting that tailored interventions could help alleviate anxiety and depression. Hernaez et al. (2022) also demonstrated that older and female cirrhosis patients are at a higher risk of depression and anxiety, reinforcing the importance of addressing mental health in these vulnerable groups. Based on these findings, incorporating regular psychological screenings and interventions into comprehensive care plans for elderly, female, and late-stage patients with HCC may be beneficial for improving their physical and mental health outcomes.

Finally, special attention should be given to the interplay between mental health and disease recurrence. Mental health is a critical component of QoL in patients with HCC. Depression and anxiety are strongly associated with impaired physical function and overall well-being, with 15–20% of patients with cirrhosis experiencing moderate to severe symptoms of these conditions (Hernaez et al. 2022). Patients receiving more invasive treatments, such as TACE, more frequently report greater symptom distress, depression, and anxiety, which highlights the importance of integrating mental health screening and support into comprehensive HCC care. As emphasized by Firkins et al. (2021), psychological counseling and support should be regarded as essential elements of care, particularly for patients with markedly reduced QoL. Disease recurrence is also linked to further declines in QoL, as patients with recurrent HCC tend to have lower functional status and higher levels of symptom distress, depression, and anxiety (Bruix et al. 2011; Fan et al. 2010). Given the limited treatment options and poorer prognosis after recurrence, early implementation of psychological assessment, counseling, and symptom management may help reduce stress, support QoL, and promote adherence to treatment. Preventive strategies should focus on early surveillance and follow-up to detect recurrence, and for patients at high risk, combining individualized treatment approaches with psychological support may be beneficial in maintaining both physical and mental health (Bonnetain et al. 2008).

### Strengths, limitations, and further research

A major strength of this study is its systematic comparison of symptom distress, psychological distress, and QoL across multiple treatment modalities for HCC, using validated measurement tools (EORTC QLQ-C30, HADS, BSRS). The study provides important insights into treatment-related differences in mental health and QoL, and highlights vulnerable subgroups such as older patients, females, and those with advanced-stage disease.

Several limitations should be acknowledged. First, the cross-sectional design restricts the ability to establish causal relationships between symptom distress, psychological distress, and QoL. Longitudinal research would be more appropriate to clarify temporal dynamics and the long-term impact of psychological distress on QoL. Second, the relatively small sample size and single-center design limit generalizability to the broader HCC population. Larger, multi-center cohorts with greater demographic diversity are needed to confirm these findings. Finally, although important associations were identified, the study did not capture biological or treatment-related biomarkers that might further explain variations in QoL.

Future studies should employ longitudinal designs to track changes in QoL over time and to identify causal pathways linking symptom distress, psychological well-being, and treatment type. Intervention studies are also warranted to evaluate tailored psychosocial and supportive care strategies, particularly for high-risk groups such as older, female, and late-stage patients. Moreover, integrating biological markers with psychosocial measures may provide a more comprehensive understanding of the mechanisms influencing QoL in HCC.

## Conclusions

This study highlights that age, gender, disease stage, recurrence status, and treatment patterns are strongly associated with variations in QoL among patients with HCC. Older individuals and those with advanced-stage disease tended to report greater physical and psychological distress, while female patients more frequently reported higher psychological burden. Patients receiving more invasive treatments, such as TACE or HAIC, also reported greater distress; however, these associations likely reflect the underlying disease severity and baseline health rather than the treatment modality alone. Minimally invasive approaches, such as RFA, remain an appropriate option for early-stage HCC in line with current guidelines, while patients undergoing more invasive or palliative therapies may benefit from closer psychological assessment and support. These findings underscore the need for comprehensive, patient-centered care strategies – combining optimal disease management, symptom control, and psychosocial support – to help maintain QoL and overall well-being throughout the disease course.

## Supporting information

10.1017/S1478951526101758.sm001 Tsai et al. supplementary material Tsai et al. supplementary material

## Data Availability

All data produced or examined in the course of this study can be found within this article and its supplementary online materials. For additional information, please contact the corresponding author.

## Supplementary material

The supplementary material for this article can be found at https://doi.org/10.1017/S1478951526101758.
