# Supplementary material for: Treatment-related differences in quality of life and psychological distress among patients with hepatocellular carcinoma: A cross-sectional analysis
Source: Palliat Support Care. 2026 Feb 5;24:e53. doi: 10.1017/S1478951526101758 (PMC13166430; doi:10.1017/S1478951526101758)
Supplement: Tsai et al. supplementary material [file S1478951526101758sup001.docx]

**Supplementary Materials**

**Materials and methods**

*Patient enrollment and study design*

A cross-sectional study was conducted from October 27, 2020, to December 31, 2021 with the approval of the Institutional Research Board of XX Hospital, XX University, Taiwan (Approval No. XXXX107-RECXXX ; approved on October 27, 2020). This study aimed to investigate the relationships among symptom distress, depression, and QoL in patients with HCC. Using a purposive sampling method, we selected 101 consecutive inpatients with confirmed HCC at a regional teaching hospital in southern Taiwan. Diagnoses were confirmed through diagnostic imaging (computed tomography or magnetic resonance imaging) and liver biopsy with histological or cytological examination.

The inclusion criteria were as follows: patients with pathological evidence of HCC or a serum α-fetoprotein (AFP) ≥ 200 ng/mL, and physician-confirmed liver cancer; patients who were aware of their liver cancer diagnosis, over 18 years of age, who were conscious and capable of communication, and who had undergone surgical or non-surgical treatment for liver cancer. Exclusion criteria were as follows: patients with esophageal variceal bleeding, gastrointestinal bleeding, hepatic encephalopathy, or alcohol withdrawal syndrome, and patients under 18 years of age, who were unconscious and unable to communicate and who had not received treatment for liver cancer.

Patients received various treatments, including hepatic resection (HR, n = 14), transarterial chemoembolization (TACE, n = 25), hepatic arterial infusion chemotherapy (HAIC, n = 8), immunotherapy (IT, n = 2), and radiofrequency ablation (RFA, n = 32). Additional treatments included symptom management (n = 13), chemotherapy (n = 1), radiotherapy (n = 2), targeted therapy (n = 3), and concurrent chemoradiotherapy (CCRT, n = 1). Symptom distress, depression, and QoL were assessed post-treatment.

A priori sample size estimation was conducted using G*Power 3.1 software for a linear multiple regression model. Assuming a medium effect size (f² = 0.15), an α level of 0.05, statistical power (1−β) of 0.80, and inclusion of up to five predictors (e.g., symptom distress, depression, treatment type, age, and disease duration), the minimum required sample size was calculated to be 92 participants. To account for potential attrition or incomplete responses, a target sample size of 100 was established. Ultimately, 101 eligible participants were enrolled and completed the study. Given the minimal risk nature of the study and the use of de-identified data, the Institutional Review Board granted a waiver of written informed consent. Nevertheless, verbal informed consent was obtained from all participants before data collection to ensure voluntary participation.

**Questionnaire**

*EORTC QLQ-C30 (version 3)*

We are interested in some things about you and your health. Please answer all the questions yourself by circling the number that best applies to you. There are no "right" or "wrong" answers. The information that you provide will remain strictly confidential.

1 = Not at All, 2= A Little, 3= Quite a Bit, 4= Very Much.

| Topic | Not at All | A Little | Quite a Bit | Very Much |
| --- | --- | --- | --- | --- |
| 1. Do you have any trouble doing strenuous activities, like carrying a heavy shopping bag or a suitcase? | 1 | 2 | 3 | 4 |
| 1. Do you have any trouble taking a long walk? | 1 | 2 | 3 | 4 |
| 1. Do you have any trouble taking a short walk outside of the house? | 1 | 2 | 3 | 4 |
| 1. Do you need to stay in bed or a chair during the day? | 1 | 2 | 3 | 4 |
| 1. Do you need help with eating, dressing, washing yourself or using the toilet? | 1 | 2 | 3 | 4 |
| **During the past week** | Not at All | A Little | Quite a Bit | Very Much |
| 1. Were you limited in doing either your work or other daily activities? | 1 | 2 | 3 | 4 |
| 1. Were you limited in pursuing your hobbies or other leisure time activities? | 1 | 2 | 3 | 4 |
| 1. Were you short of breath? | 1 | 2 | 3 | 4 |
| 1. Have you had pain? | 1 | 2 | 3 | 4 |
| 1. Did you need to rest? | 1 | 2 | 3 | 4 |
| 1. Have you had trouble sleeping? | 1 | 2 | 3 | 4 |
| 1. Have you felt weak? | 1 | 2 | 3 | 4 |
| 1. Have you lacked appetite? | 1 | 2 | 3 | 4 |
| 1. Have you felt nauseated? | 1 | 2 | 3 | 4 |
| 1. Have you vomited? | 1 | 2 | 3 | 4 |
| 1. Have you been constipated? | 1 | 2 | 3 | 4 |
| 1. Have you had diarrhea? | 1 | 2 | 3 | 4 |
| 1. Were you tired? | 1 | 2 | 3 | 4 |
| 1. Did pain interfere with your daily activities? | 1 | 2 | 3 | 4 |
| 1. Have you had difficulty in concentrating on things, like reading a newspaper or watching television?。 | 1 | 2 | 3 | 4 |
| 1. Did you feel tense? | 1 | 2 | 3 | 4 |
| 1. Did you worry? | 1 | 2 | 3 | 4 |
| 1. Did you feel irritable? | 1 | 2 | 3 | 4 |
| 1. Did you feel depressed? | 1 | 2 | 3 | 4 |
| 1. Have you had difficulty remembering things? | 1 | 2 | 3 | 4 |
| 1. Has your physical condition or medical treatment interfered with your family life? | 1 | 2 | 3 | 4 |
| 1. Has your physical condition or medical treatment interfered with your social activities? | 1 | 2 | 3 | 4 |
| 1. Has your physical condition or medical treatment caused you financial difficulties? | 1 | 2 | 3 | 4 |

For the following questions please circle the number between 1 and 7 that best applies to you.

1. How would you rate your overall health during the past week?

| Very poor |  |  |  |  |  | Excellent |
| --- | --- | --- | --- | --- | --- | --- |
| 1 | 2 | 3 | 4 | 5 | 6 | 7 |

1. How would you rate your overall quality of life during the past week?

| Very poor |  |  |  |  |  | Excellent |
| --- | --- | --- | --- | --- | --- | --- |
| 1 | 2 | 3 | 4 | 5 | 6 | 7 |

**Hospital Anxiety and Depression Scale (HADS)**

After reading each question, please circle the most appropriate answer based on your situation, using a scale from 1 to 4.

| Topic | Options | | | |
| --- | --- | --- | --- | --- |
| 1. I feel nervous (or distressed). | Almost all the time | Most of the time | Sometimes | Not at all |
|  | 1 | 2 | 3 | 4 |
| 1. I am still interested in things I used to enjoy. | Definitely the same | Not as much as before | Only a little | Hardly at all |
|  | 1 | 2 | 3 | 4 |
| 1. I feel somewhat afraid, as if something terrible is about to   happen. | Very much so and quite severe | Yes, but not too severe | A little, but it doesn't bother me | Not at all |
|  | 1 | 2 | 3 | 4 |
| 1. I can laugh out loud and see the positive side of things. | I do this often | I don’t do this as much now | I definitely do this much less now | Not at all |
|  | 1 | 2 | 3 | 4 |
| 1. My mind is filled with worries. | Most of the time | Often | Sometimes, but not frequently | Occasionally |
|  | 1 | 2 | 3 | 4 |
| 1. I feel happy. | Most of the time | Not often | Sometimes | Most of the time |
|  | 1 | 2 | 3 | 4 |
| 1. I can sit comfortably and relax. | Definitely | Often | Not often | Not at all |
|  | 1 | 2 | 3 | 4 |
| 1. I have lost interest in my appearance (taking care of myself). | Definitely | I am not as concerned as I should be | I may not be very concerned | I am as concerned as I’ve always been |
|  | 1 | 2 | 3 | 4 |
| 1. I feel somewhat restless, as if I need to be constantly moving. | Definitely | I am not as concerned as I should be | I may not be very concerned | I am as concerned as I’ve always been |
|  | 1 | 2 | 3 | 4 |
| 1. I look at everything with optimism. | Almost always | Not completely | Rarely | Almost never |
|  | 1 | 2 | 3 | 4 |
| 1. I suddenly experience feelings of panic. | Very often | Often | Not often | Not at all |
|  | 1 | 2 | 3 | 4 |
| 1. I feel like my mood is gradually getting lower. | Almost all the time | Very often | Sometimes | Not at all |
|  | 1 | 2 | 3 | 4 |
| 1. I feel somewhat afraid, as if one of my internal organs is malfunctioning. | Not at all | Sometimes | Very often | Almost all the time |
|  | 1 | 2 | 3 | 4 |
| 1. I can enjoy a good book or a quality radio or TV program. | Often | Sometimes | Not often | Rarely |
|  | 1 | 2 | 3 | 4 |

**Brief Symptom Rating Scale (BSRS-5)**

Please carefully reflect on the past week (including today) and select the answer that best represents how troubled or distressed you felt about these issues on a scale from 1 to 5.

0 = Not at all, 1 = Mild, 2 = Moderate, 3 = Severe, 4 = Very severe.

| Topic | Not at all | Mild | Moderate | Severe | Very severe |
| --- | --- | --- | --- | --- | --- |
| 1. Difficulty sleeping, such as trouble falling asleep, waking up easily, or waking up too early. | 0 | 1 | 2 | 3 | 4 |
| 1. Feeling nervous or restless. | 0 | 1 | 2 | 3 | 4 |
| 1. Feeling easily upset or irritable. | 0 | 1 | 2 | 3 | 4 |
| 1. Feeling depressed or down. | 0 | 1 | 2 | 3 | 4 |
| 1. Feeling inferior to others. | 0 | 1 | 2 | 3 | 4 |
| Total score for questions 1–5  ⬜ 0〜5 points ⬜ 6〜9 points ⬜ 10〜14 points ⬜ 15 points or more Total score for | | | | | |
| 1. Having thoughts of suicide. | 0 | 1 | 2 | 3 | 4 |

**(1) Total score for questions 1 to 5:**

Score of 0–5: Falls within the normal range, indicating good mental and physical well-being.

Score of 6–9: Mild emotional distress. It is recommended to talk to family or friends to relieve emotions.

Score of 10–14: Moderate emotional distress. Seeking counseling or professional advice is recommended.

Score of >15: Severe emotional distress. Professional counseling or treatment by a psychiatrist is strongly advised.

**(2) Scoring for question 6:** If the total score for questions 1 to 5 is less than 6, but the score for question 6 is 2 or higher (moderate), psychiatric consultation should be considered.

**Supplementary Table S1** Correlation analysis of depression and anxiety, BSRS, and QoL in HR patients.

| Variables | QoL (EORTC-QLQ-C30) | | | Depression and anxiety  (HADS) | Psychological distress (BSRS) |
| --- | --- | --- | --- | --- | --- |
|  | Global health status | Function | Symptom |  |  |
| QoL (EORTC-QLQ-C30) |  |  |  |  |  |
| Global health status | - | - | - | - | - |
| Function | 0.77^b^ | - | - | - | - |
| Symptom | -0.74^b^ | -0.67^b^ | - | - | - |
| Depression and anxiety  (HADS) | -0.67^a^ | -0.72^b^ | 0.72^b^ | - | - |
| Psychological distress (BSRS) | -0.68 ^a^ | -0.90^c^ | 0.79^c^ | 0.74^b^ | - |

Note: Significance levels were indicated as follows: ^a^ *p*< .05, ^b^ *p* < .01, ^c^ *p* < .001. QoL: Quality of Life. HADS: Hospital Anxiety and Depression Scale. BSRS: Brief Symptom Rating Scale.

**Supplementary Table S2** Correlation analysis of depression and anxiety, BSRS, and QoL in TACE/HAIC/IT patients.

| Variables | QoL (EORTC-QLQ-C30) | | | Depression and anxiety  (HADS) | Psychological distress (BSRS) |
| --- | --- | --- | --- | --- | --- |
|  | Global health status | Function | Symptom |  |  |
| QoL (EORTC-QLQ-C30) |  |  |  |  |  |
| Global health status | - | - | - | - | - |
| Function | 0.66 | - | - | - | - |
| Symptom | -0.60 | -0.85 | - | - | - |
| Depression and anxiety  (HADS) | -0.67 | -0.78 | 0.71 | - | - |
| Psychological distress (BSRS) | -0.64 | -0.89 | 0.79 | 0.80 | - |

Note: All correlation coefficients were statistically significant at *p* < .001. QoL: Quality of Life. HADS: Hospital Anxiety and Depression Scale. BSRS: Brief Symptom Rating Scale.

**Supplementary Table S3** Correlation analysis of depression and anxiety, BSRS, and QoL in RFA patients.

| Variables | QoL (EORTC-QLQ-C30) | | | Depression and anxiety  (HADS) | Psychological distress (BSRS) |
| --- | --- | --- | --- | --- | --- |
|  | Global health status | Function | Symptom |  |  |
| QoL (EORTC-QLQ-C30) |  |  |  |  |  |
| Global health status | - | - | - | - | - |
| Function | 0.54^a^ | - | - | - | - |
| Symptom | -0.59^b^ | -0.64^b^ | - | - | - |
| Depression and anxiety  (HADS) | -0.51^a^ | -0.74^b^ | 0.56^a^ | - | - |
| Psychological distress (BSRS) | -0.48^a^ | -0.81^b^ | 0.66^b^ | 0.74^b^ | - |

Note: Significance levels were indicated as follows: ^a^ *p* < .01, ^b^ *p* < .001. QoL: Quality of Life. HADS: Hospital Anxiety and Depression Scale. BSRS: Brief Symptom Rating Scale.
